# Advocacy for Increased International Efforts for Antimicrobial Stewardship Actions in Low-and Middle-Income Countries on Behalf of Alliance for the Prudent Use of Antimicrobials (APUA), Under the Auspices of the International Society of Antimicrobial Chemotherapy (ISAC)

**DOI:** 10.3389/fmed.2020.00503

**Published:** 2020-08-25

**Authors:** Pierre Tattevin, Gabriel Levy Hara, Adnene Toumi, Mushira Enani, Geoffrey Coombs, Andreas Voss, Heiman Wertheim, Armel Poda, Ziad Daoud, Ramanan Laxminarayan, Dilip Nathwani, Ian Gould

**Affiliations:** ^1^Infectious Diseases and Intensive Care Unit, Pontchaillou University Hospital Center, Rennes, France; ^2^Infectious Diseases Unit, Hospital Carlos G. Durand, Buenos Aires, Argentina; ^3^Infectious Diseases Department, Monastir University Hospital, Monastir, Tunisia; ^4^Faculty of Medicine, King Fahad Medical City, Riyadh, Saudi Arabia; ^5^Antimicrobial Resistance and Infectious Diseases Research Laboratory, Murdoch University, Perth, WA, Australia; ^6^Department of Medical Microbiology and Infectious Diseases, Canisius-Wilhelmina Hospital, Nijmegen, Netherlands; ^7^Radboudumc, Department of Medical Microbiology and Radboud Center for Infectious Diseases, Nijmegen, Netherlands; ^8^Infectious Diseases Department, Sourô Sanou University Hospital, Bobo-Dioulasso, Burkina Faso; ^9^Clinical Microbiology, Saint George Hospital-UMC and University of Balamand, Beirut, Lebanon; ^10^Center for Disease Dynamics, Economics and Policy (CDDEP), New Delhi, India; ^11^Ninewells Hospital and Medical School, Dundee, United Kingdom; ^12^Aberdeen Royal Infirmary, Aberdeen, United Kingdom

**Keywords:** antimicrobial stewardship, low- and middle- income countries, antibiotics, resistance, education

## Abstract

Antimicrobial stewardship (AMS) is a set of coordinated strategies to improve the use of antimicrobials, to enhance patient outcomes, reduce antimicrobial resistance, and decrease unnecessary costs. The pioneer years of AMS were restricted to high-income countries (HIC), where overconsumption of antibiotics was associated with emergence of multidrug-resistant (MDR) bacteria. AMS in low- and middle-income countries (LMIC) is also necessary. However, programs effective in HIC may not perform as well in LMIC, because (i) While decreased consumption of antibiotics may be an appropriate target in overconsuming HIC, this may be dangerous in LMIC, where many patients die from the lack of access to antibiotics; (ii) although AMS programs in HIC can be designed and monitored through laboratory surveillance of resistance, surveillance programs are not available in many LMIC; (iii) the heterogeneity of health care systems implies that AMS programs must be carefully contextualized. Despite the need to individually tailor AMS programs in LMIC, international collaborations remain highly valuable, through the dissemination of high-quality documents and educational material, that may be shared, adapted where needed, and adopted worldwide. This process, facilitated by modern communication tools, combines many benefits, including: (i) saving time, a precious dimension for health care workers, by avoiding the duplication of similar works in different settings; (ii) taking advantage of colleagues skills, and initiatives, through open access to the work performed in other parts of the world; (iii) sharing experiences, so that we all learn from each others' successes and failures.

## Introduction

According to the World Bank, low-income countries are defined as those with a global national income (GNI) per capita, of ≤ 1,025 US$ (2018 data for the fiscal year 2020), lower middle-income countries are those with a GNI per capita between 1,026 and 3,995 US$, and upper middle-income countries are those with a GNI per capita between 3,996 and 12,375 US$. These countries are gathered under the category “low- and middle-income countries” (LMIC), despite large heterogeneities between them, not only in terms of resources, but also in terms of health care systems, access to care, or the human development index (1). Despite the caveats of this definition, LMIC is often used to refer to countries where resources constraints are associated with poor health, and a greater need to prioritize health care interventions that will target the diseases with the highest impact on public health, and be cost-effective (2). In the context of infectious diseases and antimicrobial resistance (AMR), LMIC in Sub-Saharan Africa and Asia carry the greatest burden. They have to contend with weak regulatory infrastructure, over-the-counter sales or counterfeit antimicrobials, and inappropriate prescription practices coupled with significant gaps in diagnostic testing and surveillance. This situation leads many to predict that AMR will disproportionally impact populations living in LMICs (3).

The term “antimicrobial stewardship” (AMS), first coined in 1996 (4), is defined as a “set of coordinated strategies to improve the use of antimicrobial medications with the goals to enhance patient health outcomes, reduce antimicrobial resistance, and decrease unnecessary costs” (5). Primarily targeting the prescribers, AMS includes not only limiting inappropriate use, but also optimizing antimicrobial selection, dosing, route, and duration of therapy to maximize its efficacy whilst limiting the unintended consequences, such as the emergence of resistance, and adverse events. Initially, AMS programs were mostly restricted to high-income countries (HIC), where overconsumption of antibiotics was increasingly associated with emergence of multidrug-resistant (MDR) bacteria, and *Clostridoides difficile* infections. In the US, the proven benefits of AMS programs have prompted the Centers for Disease Control and Prevention (CDC) to recommend that all hospitals have an AMS program, with defined core elements (6). The CDC recommendation has recently been redefined on a global scale (7). Although the global volume of antibiotic use is 5–15 times higher outside, than inside hospitals, and has been linked to the development of AMR in the community (8), AMS programs for the community use of antibiotics have paradoxically remained neglected. We therefore need to prioritize AMS in the community setting. For example, the work being undertaken in the UK (9) gives us some insight into what is possible. There needs to be an integrated one-system whole-health-economy approach to AMS, as recently advocated (10). This whole system approach is likely to be more effective and sustainable, compared to the fragmented or piece meal primarily hospital-focused approach being delivered in most countries. Indeed, one example from Zambia illustrates what is possible, and the attendant impact in a LMIC setting (11).

Implementation of hospital AMS programs in LMIC is challenging (12), and even more so in the primary care or community setting. For example, in 2014, despite over three quarters of the member countries of the WHO Regional Office of Africa (AFRO) having a medicine policy emphasizing rational use of antibiotics, the majority of countries have limited capacity to effectively implement these policies, particularly in primary health care. The poor implementation of antibiotic policies in most LMIC is attributed to the lack of enforcement of the available polices and systems as well as the unavailability of surveillance data and laboratory capacity for monitoring antibiotic resistance (13).

It is therefore imperative that AMS interventions across all systems are effectively implemented, given that MDR is a one-system and one-health worldwide issue that won't be controlled without global intervention (14–16). The need for these global interventions has been well-illustrated by the “One Health” concept that includes not only the prescribers and the patients (14), whether in the hospital or in the community, but also animals and the environment. Of note, the tools available to reduce the burden of antibiotic resistance should not be restricted to AMS, as vaccines (17), infection control programs, and improved sanitation (18), have all demonstrated their benefits to reduce the emergence of MDR bacteria, when adequately implemented at a large scale.

## The Specific Points to Condiser for AMS Programs in LMIC

There are several reasons why proven AMS programs that were proven effective in HIC may not perform as well in LMIC, and consequently their implementation “as it is” would provide disappointing outcomes (19, 20). The main differences that must be taken into account for the design and implementation of AMS programs in LMIC, starting from the experience gained in HIC, are summarized in Table 1. Firstly, while most HIC have documented overconsumption of antibiotics, with limited exceptions [namely, the Netherlands, and Scandinavian countries, in Europe (21)], this may not be the case for most LMIC. Actually, there are robust data to suggest that worldwide, more patients die because they don't have access to appropriate antibiotics for easy-to-treat infections (e.g., pneumonia), than because of antimicrobial resistance (22). Hence, while AMS programs in HIC could safely target decreased consumption of antibiotics, through the reduction of inappropriate use of antibiotic, such targets may be dangerous in LMIC, where increased access to appropriate antibiotics when needed should be one of the top priorities (23). Although this may sound provocative, a dramatic increase in antibiotics consumption, as recently demonstrated in many LMIC (24), may indeed be good news, as it may translate in millions of lives saved, if these antibiotics are appropriately used. However, this positive impact is definitely not guaranteed, due to the sub-optimal qualification of health care workers and the limited technical platform to guide diagnoses in LMIC. Of note, global point prevalence survey showed that around 35% of antimicrobials are misused in hospitals from LMIC in Latin America, Africa, and Asia.

**Table 1:** Differences to take into account for the design of antimicrobial stewardship programs in low- and middle-income countries, as compared to high-income countries.

	**High-income countries**	**Low- and middle- income countries**
**Use of antibiotics (24)**		
**- quantity**	25 defined daily doses (DDD) per 1,000 inhabitants per day	10 (low- and lower-middle-income countries), to 20 (higher-middle-income countries) DDD per 1,000 inhabitants per day
**- trends 2000–2015**	Steady	Increasing (+77%)
**- quality**	Regular monitoring	Limited or no surveillance
**Basic laboratory services with access to data on antimicrobial resistance in human pathogens**	Routinely available for individual patients, and periodic surveillance in different settings (hospitals, community)	Limited or no data
**Procurement of antibiotics**	Only if prescribed by medical doctors, with rare exceptions	Highly heterogeneous, but often available through the prescription of various health care workers, and even without prescription in many settings (street vendors)
**Human resources available for antimicrobial stewardship actions**	Heterogeneous, and insufficient in most countries, but increasing	Close to zero in many countries often not considered as a priority
**Education and training for health care workers on antimicrobial resistance and use**	Usually more extensive, both in pre-service as well as in-service	Less time dedicated in pre-service schools suboptimal in post-graduate settings, due to lack of prioritization, human restrained resources, and background

Secondly, in HIC, AMS programs are designed and monitored, through laboratory surveillance of antimicrobial resistance in inpatients, and outpatients. Sadly, the current status of the diagnostic microbiology laboratory in LMIC would very rarely allow such support for AMS programs (25). Although point-prevalence surveys have demonstrated MDR bacteria have emerged in many LMIC (26–28), with striking heterogeneity, in humans as well as in animals (29), such data remain scarce: Many colleagues in LMIC work in an environment where they have no, or limited information about the prevalence of resistance to the antibiotics they use in common situations. Even in countries where surveillance is in place, updated, communication to prescribers is sub-optimal. In most cases, there are no official channels to disseminate the information, and prescribers do not usually check for updated AMR data. This lack of baseline information jeopardizes the implementation of appropriate AMS programs, and the monitoring of their impact over time (30).

Thirdly, the architecture of the health care systems, broadly heterogeneous in LMIC, must be cautiously evaluated and taken into account before AMS programs are initiated. Even well-planned interventions may have no benefit if they are implemented in structures where patients rarely go. The contextualization of AMS programs in LMIC is of paramount importance, as inappropriate programs may even be deleterious, in many aspects: (i) restriction of antibiotic use may be effective and safe in countries with broad access to diagnostic tests, and the possibility to closely monitor patients, but is more risky in LMIC where such possibilities often don't exist; (ii) although prescription of antibiotics is limited to trained physicians in most HIC, this is not the case in many LMIC, consequently training limited to physicians may have limited impact, if patients have easy access to antibiotics through other providers, including various health care workers with limited or no training on AMS, or even street vendors. Actually, task shifting from medical doctors to non-specialist pharmacists and nurses for antibiotic prescription may be feasible, and effective, but this has to be contextualized (31, 32).

Culture is defined as the knowledge that people use to develop shared beliefs, practices and norms that distinguish one group of people from another, e.g., the culture across different specialties, organizations or countries can influence and shape behaviors and intervention outcomes. In the context of implementing AMS programs this has been identified as a key barrier to effective stewardship. A recent study across HIC and LMIC documented a culture of hierarchies that dominated the effectiveness and reach of AMS programs. For example, professional boundaries limited the involvement of nursing and pharmacy staff with doctors remaining the key stakeholders. An understanding of this could provide solutions such as AMS champions and local leadership that can be used to overcome hierarchical and rigid national and organizational cultures (33).

Education and training in AMR is without doubt a keystone to optimize the use of diagnostic tools, and for improving the use of antimicrobials. By no means restricted to LMICs, the issues of inadequate education and training in these settings may be bigger both in pre and in-service. The scarcity of definition of objectives and minimum contents to be taught and the lack of harmonization between different universities in the same country generate important gaps in knowledge. Regarding in-service, there are less opportunities in LMICs for high-level training than in HIC. By November 2019, WHO launched a curricula guide for health worker's education and training in AMR (34). This new tool includes a systematic modular and submodular collection of learning objectives and outcomes organized according to the key occupational groups involved in the use of antimicrobials in human health. The occupational groups covered include prescribers of antimicrobials, nurses, midwives, pharmacists, laboratory scientists, public health officers, and health services managers. The goal of the curricula guide is to provide the practical competencies to manage antimicrobials according to their roles.

## Antimicrobial Stewardship Within the Global Action Plan for Antimicrobial Resistance, and Other International Initiatives

In 2015, the World Health Organization (WHO) released a global action plan on antimicrobial resistance (http://apps.who.int/gb/ebwha/pdf_files/WHA68/A68_20-en.pdf), with five strategic objectives: (i) to improve awareness and understanding of antimicrobial resistance; (ii) to strengthen knowledge through surveillance and research; (iii) to reduce the incidence of infection; (iv) to optimize the use of antimicrobial agents; and (v) to ensure sustainable investment in countering antimicrobial resistance. The objective (iv) clearly encompasses to AMS, with the following recommendation to member states: “*Provision of stewardship programs that monitor and promote optimization of antimicrobial use at national and local levels in accordance with international standards in order to ensure the correct choice of medicine at the right dose on the basis of evidence*”. The WHO explicitly encourages “*implementation of AMS programs with international and national partners across multiple sectors, accompanied by actions to ensure affordable and equitable access by those who need them”* (35). In line with this call, two recommendations for implementing AMS programs were launched. By November 2018, the Pan-American Health Organization (PAHO/WHO), jointly with the Global Health Consortium at the Florida International University (GHC/FIU) published the “Recommendations for Implementing AMS Programs in Latin America and the Caribbean,” including both primary care settings and hospitals (36). In November 2019, WHO published a practical toolkit for implementing AMS programs in LMIC hospitals (37). The assets of local, regional, and international collaboration with examples of good practices or success stories were recently highlighted (38).

**Figure 1:**
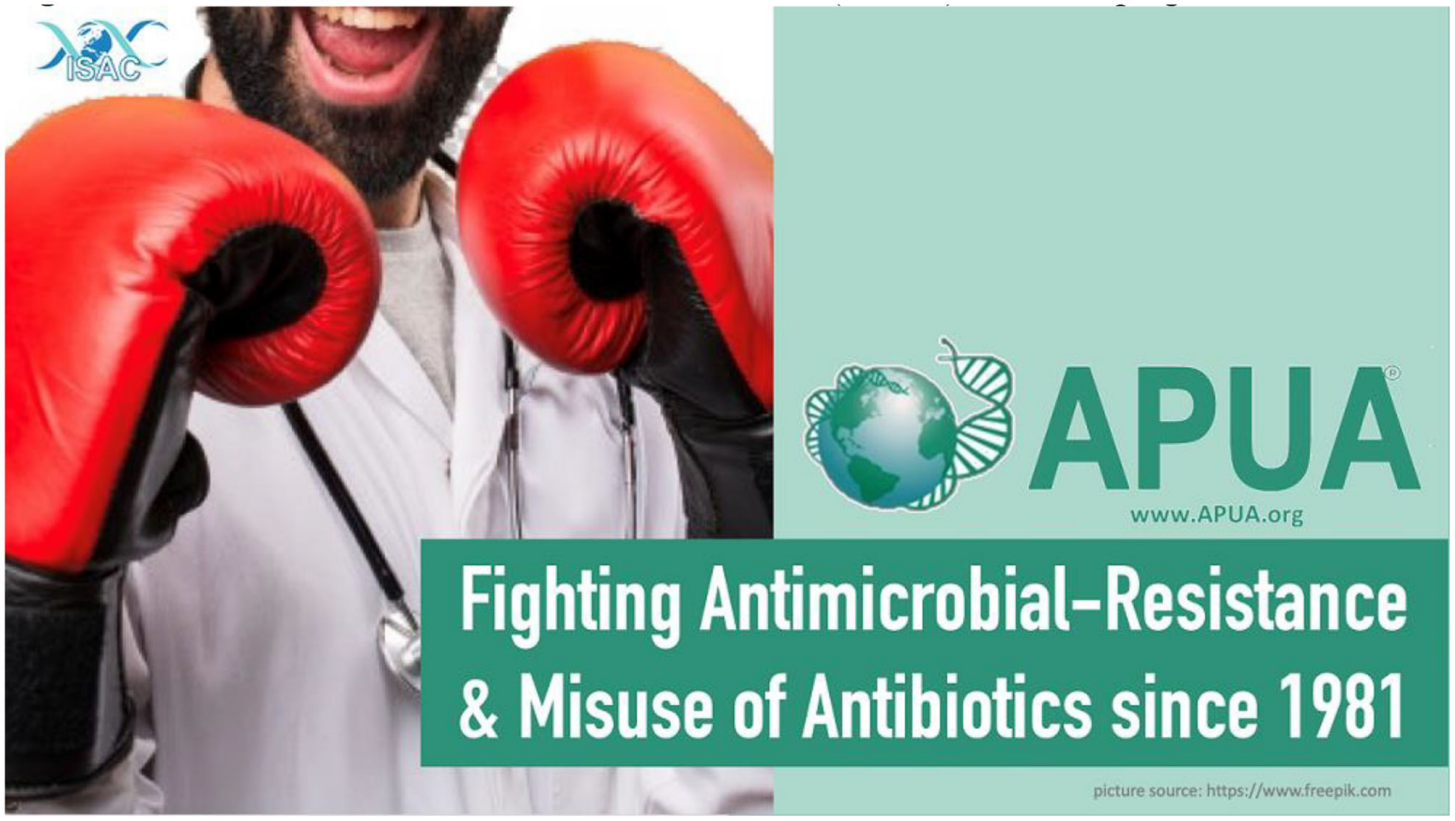
Alliance for the Prudent Use of Antibiotics (APUA) 2019 campaign.

Five years after the launch of WHO global action plan, many who have worked in the field have the feeling that, although progress have been achieved (12, 39, 40), with remarkable success stories (41–43), the actions undertaken to optimize the use of antimicrobial agents do not match the challenges of emerging antimicrobial resistance in most countries (23). In addition, the durability of progress achieved in AMS requires sustained efforts, otherwise the benefits may be rapidly lost (30, 43). In line with the WHO global action plan, there are various initiatives led by professional societies or non-governmental bodies, aimed at reducing the emergence of antimicrobial resistance through international collaborations over the last decades. Some are described below:

- The Alliance for the Prudent Use of Antibiotics (APUA), was founded in 1981 by Stuart Levy, one of the pioneers in the fight against antimicrobial resistance, to “*maximise the effectiveness of antimicrobial treatment by promoting appropriate antimicrobial use and containing drug resistance”*. With a broad international network of “chapters” in countries from Africa, America, Asia, Europe and Oceania, APUA has long been a major platform exchange, and facilitator of multi-sectorial actions, from education to advocacy, targeting the lay population as well as health care workers and politicians (Figure 1). APUA merged with the AMS Working Group of the International Society of Antimicrobial Chemotherapy (ISAC) in 2019, to join forces for the promotion of better use of antibiotics (https://apua.org/). A recent example of APUA collaboration was the British Society for Antimicrobial Chemotherapy (BSAC) highly impactful AMS collaboration in the Middle East and Africa regions (https://apua.org/apua-newsletter).
- ReAct, was initiated in 2005, with the goal to be a global catalyst, advocating and stimulating for global engagement on antimicrobial resistance by collaborating with a broad range of organizations, individuals and stakeholders. It offers open access to a broad range of information, and tools, to support action against antimicrobial resistance worldwide. ReAct was one of the first international independent networks to articulate the complex nature of antimicrobial resistance and its drivers (https://www.reactgroup.org/).
- The Global Antibiotic Resistance Partnership (GARP), initiated in 2009, is a collaborative platform for developing actionable policy proposals on antimicrobial resistance (https://cddep.org/partners/global-antibiotic-resistance-partnership/).
- In September 2016, the United Nations (UN) issued the “*declaration of the high-level meeting of the General Assembly on antimicrobial resistance*,” (https://www.who.int/antimicrobial-resistance/events/UNGA-meeting-amr-sept2016/en/), where nation leaders from all over the world committed to fighting antimicrobial resistance together. This was only the fourth time in the history of the UN that a health topic was discussed at the General Assembly (HIV, non-communicable diseases, and Ebola were the others), which illustrates the seriousness and scope of the situation. Participating nations agreed on sustainable, multi-sectoral approaches to addressing antimicrobial resistance.
- Following the 2016 UN declaration, the Conscience of Antimicrobial Resistance Accountability (CARA) initiative was launched to monitor what is done to preserve the effectiveness of antibiotics in every country on earth (https://cddep.org/blog/posts/cara_conscience_antimicrobial_resistance_accountability_and_next_big_thing_cddep/). CARA was intended as “*the eyes and ears of the world to track what is actually being done, in service to the global leadership that will lead the way following the UN General Assembly”*.

## To Start With Success: The Low Hanging Fruits

In front of the daunting task to fight the worldwide emergence of antimicrobial resistance, it seems reasonable to target the “low hanging fruits,” i.e., the actions that may be undertaken with limited resources, in terms of funding and/or staff. These principles have been applied with success in HIC as well, where AMS has long remained neglected (and still is, in many aspects). Hence, pioneer actions had to get started with no or limited dedicated funding. For example, to reduce the duration of antibacterial treatment for common infections has proven to be quite easy to implement through training of prescribers, with dissemination of guidelines, supported by randomized trials that demonstrated the equivalent efficacy of short regimens for community-acquired pneumonia, pyelonephritis, intra-abdominal infections and other conditions. This has been one of the most popular “low hanging fruits” for AMS teams in the hospitals, and even in the community. It has been shown that one can decrease by at least 30% the volume of antibiotic prescription for common infections just by convincing hospital colleagues to adjust the duration of treatment to what is needed, with no additional risk of failure (44). On the other hands, efforts to reduce the empirical use of antibiotics in patients with suspicion of sepsis, or in patients with fever in the hematology wards, is more complicated and would require much more investment before the fruits will be picked, due to the limited evidence behind the decision to prescribe, or not to prescribe, antibiotics in those patients. Indeed, failure to initiate antibiotics in those patients in case of need may have severe consequences, while patients who really require antibiotics may be difficult to identify.

Among the low hanging fruits identified for AMS actions within LMIC, dissemination of educational material through internet may be one of the most efficient and cost-effective. This, of course, requires careful consideration of the objectives, and the targets, as the messages must be adapted to the context. Several initiatives have shown that clever use of modern communication tools may indeed disseminate educational material, about the importance of careful use of antimicrobial agents:

- The e-bug initiative, launched in 2006 by Public Health England, with initial co-funding by the European Commission Directorate General for Health and Consumers, currently involves a consortium of 28 partner's countries. The main aim of the project is to educate school children across the globe, about microbiology, hygiene and the spread, treatment and prevention of infectious diseases. e-Bug also aims to reinforce an awareness of the benefits of prudent antibiotic use and how inappropriate use can have an adverse effect on antimicrobial resistance in the community (45). e-Bug provides free online educational material with separate packs for teachers, and students, including interactive lesson plans, complementary games, and quizzes (https://e-bug.eu/).
- The Multidisciplinary French course on Antimicrobial Stewardship in Africa (MUFASA) project was initiated in 2016 by Nazi Boni Universty (Burkina Faso), and Montpellier University (France). The objectives are to raise awareness and provide basic skills on AMS to 50–55 health care workers from 15 to 20 sub-Saharan African countries during 5 consecutive weeks each year, with a consortium of international partners, including WHO, the West African Health Organization (WAHO), Fondation Mérieux, and the Société de Pathologie Infectieuse de Langue Française (French Society of Infectious Diseases, SPILF): http://www.diu-antibio.org. This has been complimented in 2019 by a Massive Open Online course (MOOC) specifically for the African context developed by BSAC in collaboration with the Infection Control Africa Network (ICAN), https://www.futurelearn.com/courses/antimicrobial-stewardship-for-africa.
- The British Society for Antimicrobial Chemotherapy (BSAC), a learned professional and charitable society, has taken an impressive global and leadership role in developing, disseminating and evaluating a range of AMR, and particularly AMS, traditional and e-learning educational resources that are high-quality, global in the their focus, and at all times open access. For example, since 2015, BSAC has published 12 open access resources (http://bsac-vle.com), with a further 6 in development that have been used by 105,000+ learners from 37,000+ locations across 182 countries, with translations into five languages, with others planned (42). To-date, the cost (less the in-kind staffing contribution from BSAC), of development, roll-out, and ongoing support, is equivalent to < $4 per learner. This is supplemented by the unique open access AMS e-book developed by 39 authors across the globe (http://bsac.org.uk/antimicrobial-stewardship-from-principles-to-practice-e-book/), and the publication of JAC-Antimicrobial Resistance (https://academic.oup.com/jacamr), an innovative education and research platform that, for the first time, offers academia and industry the opportunity to submit their educational resources for peer review and publication, allowing healthcare workers a global access to peer-reviewed quality assured educational resource through this and an associated database of WHO indexed non-peer review free resources from across the globe (http://bsac-jac-amr.com/jac-amr-resources/). A library of additional e- resources is also available on the new infection management learning hub—http://www.infectionlearninghub.co.uk.

## Conclusions

The characteristics of antimicrobial use in LMIC are heterogeneous, and must be taken into account when designing any intervention that aims to maximize the effectiveness of antimicrobial treatment, by promoting appropriate antimicrobial use and containing drug resistance. The advent of antibiotics almost one century ago has dramatically improved the prognosis of most severe bacterial infections, which can still save millions of lives, provided (i) severe bacterial infections are diagnosed, and managed, in a timely manner; (ii) patients in need have access to effective antibiotics; (iii) inappropriate use of antibiotics is reduced, so that the emergence of antimicrobial resistance does not jeopardize the success of antibiotics currently available.

Despite the need to tailor AMS programs in the LMIC, international collaborations remain highly valuable, through the dissemination of high-quality documents, clinical research and educational resources, that may be shared worldwide. This process, which is relatively easy with modern digital communication tools, combines many advantages, including: (i) saving time, a precious dimension for health care workers, by avoiding the duplication of similar works in different settings worldwide; (ii) utilizing colleagues skills and initiatives, through open access to their work performed in other parts of the world; (iii) sharing experiences, so that we all improve and learn from others successes and failures.

## Author Contributions

PT wrote the first draft of the manuscript. DN, RL, GL, AT, ME, GC, AV, HW, AP, ZD, and IG reviewed the first draft and the final manuscript and provided critical comments. All authors contributed to the article and approved the submitted version.

## Conflict of Interest

The authors declare that the research was conducted in the absence of any commercial or financial relationships that could be construed as a potential conflict of interest.
